# MXenes-Based Bioanalytical Sensors: Design, Characterization, and Applications

**DOI:** 10.3390/s20185434

**Published:** 2020-09-22

**Authors:** Reem Khan, Silvana Andreescu

**Affiliations:** Department of Chemistry and Biomolecular Science, Clarkson University, Potsdam, New York, NY 13676, USA; rekhan@clarkson.edu

**Keywords:** MXenes, 2D nanomaterials, biosensors, wearables

## Abstract

MXenes are recently developed 2D layered nanomaterials that provide unique capabilities for bioanalytical applications. These include high metallic conductivity, large surface area, hydrophilicity, high ion transport properties, low diffusion barrier, biocompatibility, and ease of surface functionalization. MXenes are composed of transition metal carbides, nitrides, or carbonitrides and have a general formula M_n+1_X_n_, where M is an early transition metal while X is carbon and/or nitrogen. Due to their unique features, MXenes have attracted significant attention in fields such as clean energy production, electronics, fuel cells, supercapacitors, and catalysis. Their composition and layered structure make MXenes attractive for biosensing applications. The high conductivity allows these materials to be used in the design of electrochemical biosensors and the multilayered configuration makes them an efficient immobilization matrix for the retention of activity of the immobilized biomolecules. These properties are applicable to many biosensing systems and applications. This review describes the progress made on the use and application of MXenes in the development of electrochemical and optical biosensors and highlights future needs and opportunities in this field. In particular, opportunities for developing wearable sensors and systems with integrated biomolecule recognition are highlighted.

## 1. Introduction

Since the discovery of graphene, two-dimensional (2D) nanomaterials have gained significant attention due to their high surface area, electrical conductivity, functionalized surfaces, and mechanical properties. Prime examples include materials such as silicene, germanene, boron nitride, and molybdenum sulfide. 2D materials offer exceptional physical, chemical, and structural properties that make them useful in a wide variety of applications such as sensors, energy storage and conversion, optoelectronics, and catalysis. MXene is an emerging class of 2D transition metal carbide, nitride, or carbonitride added to the 2D nanomaterial group initially developed by Gogotsi and coworkers in 2011 [1]. MXenes have general formula M_n+1_X_n_ where M is an early transition metal while X is carbon and/or nitrogen, synthesized by the selective etching of MAX phases [2]. MAX phases are layered ternary carbides and nitrides with a general formula M_n+1_AX_n_, where A represents elements from the group 13 and 14 of the periodic table. The term MAX phases were established in the late 1990s, but most of these phases were discovered 40 years ago. A renewed interest in the application of MAX phases started in 1996 due to their unusual combination of chemical, physical, electric, and mechanical properties resulting from their layered structure supported by a strong mixed metallic-covalent *M X* bond and a relatively weak *M A* bond [3].

To date, 70 different MAX phases have been reported, while more than 20 members of the MXenes family have been synthesized, and dozens more are predicted, making it one of the fastest-growing 2D material families. Typically, 2D materials MXene are synthesized by removing the A layer from their parent MAX (remaining MX-) phases and are structurally similar with graphene (hence named -ene). Additionally, the MXene family comes in three atomic structures, ranging from M_2_X to M_3_X_2_ and M_4_X_3_, yielding tunability, and opportunity to discover and mold materials based on application requirements. Recently, the MXene family has been expanded to include double transition metal MXenes with formula M′_2_M″C_2_ and M′_2_M″_2_C_3_.

**Figure 1:**
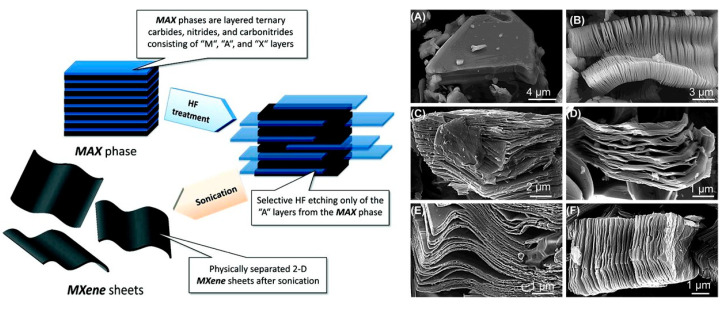
(Left) Schematic for the exfoliation process of MAX phases and formation of MXenes. (right) SEM micrographs for (**A**) Ti_3_AlC_2_ MAX phases, (**B**) Ti_3_AlC_2_ after HF treatment, (**C**) Ti_2_AlC after HF treatment, (**D**) Ta_4_AlC_3_ after HF treatment, (**E**) TiNbAlC after HF treatment, and (**F**) Ti_3_AlCN after HF treatment. Reprint with permission [2]. Copyright © 2012, American Chemical Society.

The most successful synthesis route of Ti_3_C2 involves the wet chemical etching in hydrofluoric (HF) acid or HF containing/forming etchants. The wet chemical route is based on three simple steps: (1) Etching, (2) delamination, and (3) intercalation. Due to the use of HF as an etchant, MXene layers are primarily terminated with F and OH/=O functional groups, abbreviated as *T_x_* to give a general formula of M_*n*+1_X_*n*_T_*x*_, *n* = 1–3. The etching is simply done by immersing the MAX phase in HF. Alhabeb et al. studied the effect of different concentrations of HF on the morphology of the resulting MXene. The results of the study have shown that Al can be etched by using HF concentration as low as five weight percent; however, for low HF concentrations, the MXene did show the characteristic accordion-like morphology that was observed in the case of 30% HF. After completing the etching process, the powder was washed thoroughly with deionized water by successive centrifugation cycles at 3500 rpm. After each cycle, the supernatant was decanted and replaced with fresh deionized water. The washing process continued until the pH of the supernatant has reached 6 to 7. Delamination is the second crucial step in the synthesis protocol that enhances the accessible surface area of the nanomaterial. In order to delaminate MXene nanosheets, the van der Waals forces between the adjacent MX sheets have to be broken. This barrier is relatively strong for MXenes (with their ~2.2 Å interlayer distance) compared to that of graphene (3.35 Å). A well-established method for the delamination of MXenes is to increase the interlayer distance through intercalation, achieved by inserting external elements (ions or molecules) in between the layers of the laminated material. Large, organic molecules, such as dimethyl sulfoxide (DMSO), isopropylamine, tetraalkylammonium hydroxides (TBAOH), were some of the first intercalants used for expanding the interlayer spacing of Ti_3_C_2_Tx MXenes synthesized with HF [4]. Figure 1 shows a general synthesis scheme for producing MXenes (left) and examples of SEM micrographs (right) displaying the ‘accordion’-like morphology that is typical of various MXenes compositions. For description of detailed synthesis methodologies, readers are referred to other literature, specifically discussing the synthesis protocol of MXenes [5,6]. Most applications of these materials to date are in energy conversion, catalysis, and electronics with emerging areas in structural, biomedical, and environmental fields [7,8,9]. The biosensing and analytical measurement field remains relatively unexplored and provides unique opportunities for future development.

Several prior works summarized the application of MXenes in chemical sensors [10], particularly gas sensors [11] and their environment-related applications [10,11,12,13]. Here we focus on the properties and suitability of MXenes as an immobilization matrix, signal transducer, and amplifier of biomolecular recognition for the design of biological sensors. The first half of the review provides an overview of the physical and chemical properties of MXenes that are of interest for the development of bioanalytical sensors. The second half discusses specific examples of MXenes-based biosensors with enzyme, antibodies, DNA, and aptamer recognition, and highlights recent developments on the use of MXenes as supporting material for wearable devices.

## 2. Properties of MXenes for Bioanalytical Sensing

2D nanomaterials such as graphene, MoS_2_, boron nitride nanosheets, either alone or in nanocomposite form, have demonstrated superior properties such as high surface area platforms for the construction of different types of chemical and biological sensors. When used in sensing design, these materials improved the performance of sensors in terms of sensitivity, selectivity, and analyte binding. However, limitations such as high hydrophobicity, low biocompatibility, low electrical conductivity, and difficulty in surface functionalization limit their incorporation in sensor design at a commercial scale. MXenes complement the emerging class of 2D materials and provide advantages for sensors design in terms of hydrophilicity, electrical conductivity, biocompatibility, and above all, ease of functionalization due to the presence of surface functional groups. Furthermore, MXene has high intercalation capacity, which is missing in other 2D nanomaterials. A comparison of the fundamental characteristics of different nanomaterials is given in Table 1.

The high electrical conductivity, thermal stability, hydrophilic nature, large interlayer spacing, high surface area, and easily tunable structure make this new family of 2D nanomaterials extremely attractive for a wide variety of applications. Because MXenes have a relatively low number of atomic layers, a single stack of MXene layers is typically less than 1 nm thickness, while the lateral dimension is in the nm to µm range, depending on the composition, synthesis, and processing steps. Moreover, by selecting double metal MXenes, it is possible to tune the valence states and relativistic spin-orbit coupling [31]. Furthermore, the MXenes have a ceramic-like nature that is responsible for their high chemical and mechanical stability. However, unlike traditional ceramics, MXenes have inherent conductivity and a significantly larger surface area imparted by the composition and layered structure of their molecular sheets made of carbides and nitrides of transition metals such as Ti. In addition, the MXene surface can be functionalized with different functional groups that provides numerous opportunities for surface state engineering, greatly extending their properties and applications. The ease of functionalization is a particularly important feature of using MXenes in the design of bioanalytical sensors that require materials with abundant functional groups at their surface. This high surface functionality, in addition to their layered structure, provides the ability to bind, protect, and retain the activity of biomolecules for specific targeting and recognition properties that are essential for bioanalytical sensing. The versatility and compositional variety make MXene an extraordinarily diverse and appealing class of materials for applications [31].

**Figure 2:**
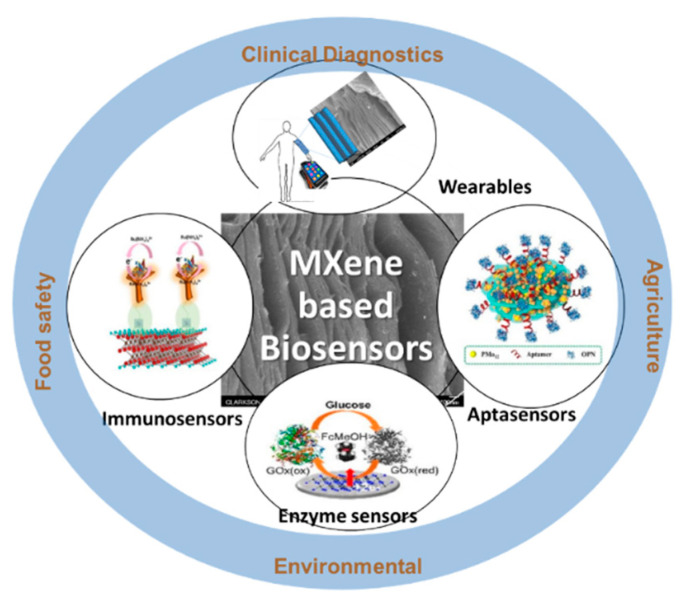
Summary of different classes of biosensing platforms based on MXenes and their applications. Immunosensors (reprinted with permission from [62]) Aptasensors (reprinted with permission from [63]), Enzyme sensors (reprinted with permission from [64]).

In the last 10 years, there has been growing interest in exploring the use of MXenes in the design of supercapacitors [32,33], transparent conductors [34,35], field-effect transistors [36], Li-ion batteries [37,38], electromagnetic interface shielders [39], catalysts [40], hybrid nanocomposites [41], fillers in polymeric composites [42], dual-responsive surfaces, purifiers, [43,44] suitable substrates for dyes [45], photocatalysts for hydrogen production [31], methane storage [46], and as well as photothermal conversion [47]. Applications in electronic [48,49], magnetic [50,51], optical [1,52,53], thermoelectric [54,55], and sensing devices [5,56] are being explored while new utilizations such as reaction media to facilitate catalytic and photocatalytic processes, hydrogen storage [57,58], and nanoscale superconductivity [59] are beginning to be investigated. Some of the MXenes are predicted to be topological insulators with large band gaps involving only d orbitals [60,61]. This review focuses on the applications of MXenes in the field of biological sensors, where MXenes have been used as an electrode material exploiting their high catalytic, high surface area, charge transport properties, and biocompatibility with biological matrices. The different classes of biosensing platforms and the applications covered in this review are summarized in Figure 2.

## 3. Classes of Bioanalytical Sensors Based on MXenes

### 3.1. Enzyme Sensors

**Figure 3:**
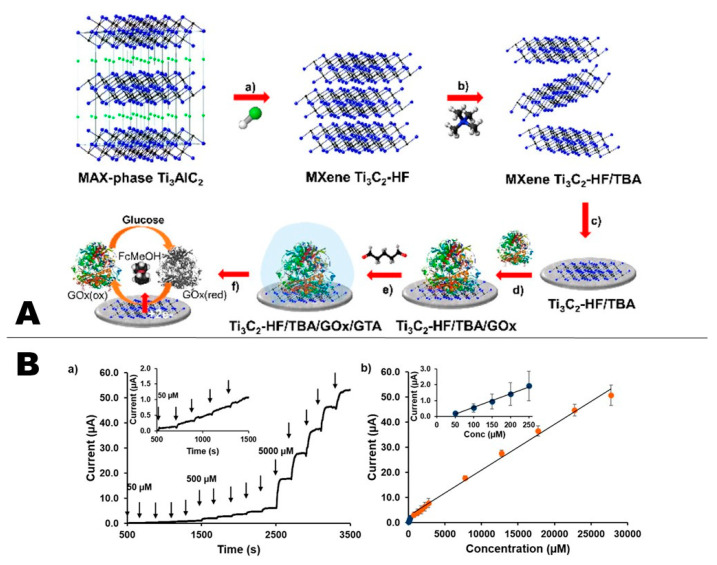
(**A**) Schematic illustration of sensor fabrication; (**a**) exfoliation of Ti_3_AlC_2_ via etching with HF; (**b**) delamination with TBAOH; (**c**) modifying the glassy carbon electrode with MXene; (**d**) loading of glucose oxidase (GOx), and; (**e**) cross-linking glutaraldehyde (GTA) with GOx; (**f**) glucose detection mechanism of the proposed biosensing system. (**B**) Chronoamperometry data (**a**) and calibration plot (**b**) for Ti_3_C_2_–HF/TBA-based electrochemical glucose biosensor conducted using FcMeOH (2 mM) in pH 7.2 PBS (electrolyte) and 0.15 V potential (reproduced with permission from reference [64]).

Enzymes, as a bio-recognition element, offer distinct advantages such as substrate specificity and high efficiency under mild conditions. Enzyme based electrochemical biosensing devices have been developed extensively in the last few decades [1]. Despite progress, enzymes can lose their bioactivity when directly immobilized onto electrode surfaces. Moreover, due to the deeply rooted location of redox-active centers in enzymes, the direct electron transfer (DET) between these biomolecules and the electrode surface is generally difficult and has been the subject of extensive investigations as reviewed in a recent work [65]. Therefore, the use of nanomaterials has been found beneficial to facilitate the electron transfer along with promoting retention of the bioactivity of immobilized enzymes. Among different types of nanomaterials, 2D nanomaterials proved to be effective for improving the DET from the enzyme to the transducer surface. Because MXene have a high surface to volume ratio and are biocompatible, they provide a highly suitable matrix for the fabrication of enzymatic biosensors. They can be used as a supporting platform for the immobilization of enzymes, promote diffusion, and accelerate the electrode kinetics, thus improving DET transfer. MXenes are also expected to enhance sensitivity and lower the detection limit of sensing devices. The most explored application of MXenes reported in the literature is for glucose sensing using glucose oxidase (GOx) immobilized within stacked layers of MXenes. For example, Chia et al. reported a Ti_3_C_2_ MXene, produced via HF etching and subsequent delamination with tetrabutylammonium hydroxide (TBAOH), as a transducer platform for the development of an electrochemical glucose biosensor with chronoamperometric detection. The biosensor exhibited high selectivity and good electrocatalytic activity toward the detection of glucose, with a linear range spanning from 50 to 750 μM and a limit of detection (LOD) of 23.0 μM (Figure 3) [64].

**Figure 4:**
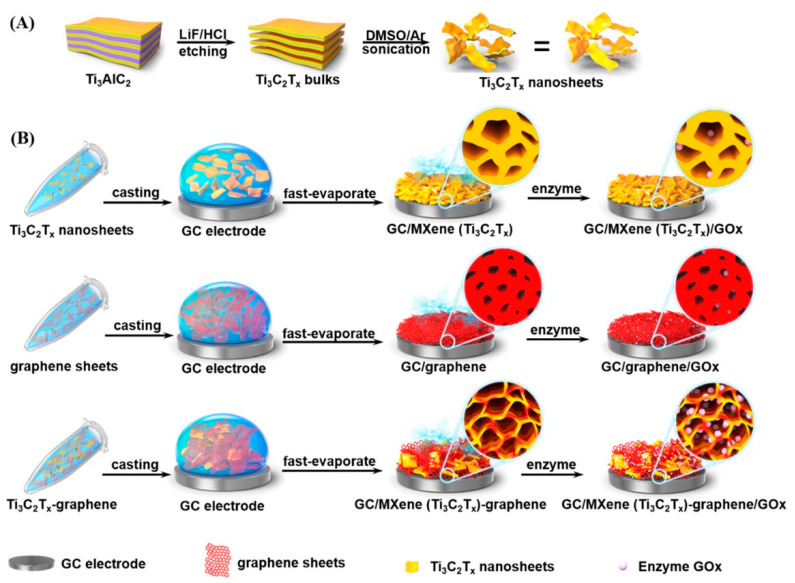
Preparation of (**A**) Ti_3_C_2_T_x_ MNS; (**B**) pure Ti_3_C_2_T_x_ film, pure graphene film, and MG hybrid film for enzyme immobilization (reproduced with permission from reference [66]).

Other works explored hybrid configurations that combine the layered structure of MXenes with the properties of other materials to add additional functions. Gu et al. constructed a porous MXene-graphene (MG) nanocomposite-based glucose biosensor [66]. The 3D porous nanostructure was prepared using a mixing-drying process in which the size of the pores was controlled by tuning the content of Ti_3_C_2_ and graphene. The synthetic methodology and the preparation process of the sensor are displayed in Figure 4. The porous structure provided more open structures to embed GOx within the internal pores, favoring retention of the GOx activity. The biosensor exhibited good electrocatalytic properties towards glucose biosensing for the detection of glucose in human sera.

Other improvements have been achieved by decorating the surface of MXenes with metal oxide nanoparticles (NPs), further increasing the surface area and conductivity of the electrodes and maximizing the enzyme loading. A composite of Nafion-Au NPs-MXene was reported as electrode material for the immobilization of GOx and subsequent detection of glucose [67]. The synergistic effects of Au NPs and MXene sheets resulted in unique electrocatalytic properties, which enabled the detection of glucose in the μM to mM range. Similarly, Ti_3_C_2_T_x_ nanosheets were modified with β-hydroxybutyrate dehydrogenase and then used as a biosensor for amperometric sensing of β-hydroxybutyrate, a biomarker for diabetic ketoacidosis. The developed biosensor best operated at a potential of −0.35 V (vs. Ag/AgCl), displayed a linear range between (0.36 to 17.9 mM), a sensitivity of 0.480 μA mM^−1^ cm^−2^, and a LOD of 45 μM. Later, the biosensor was successfully applied to the determination of β-hydroxybutyrate in (spiked) real serum samples [68].

Wang et al. reported a mediator free biosensor for the detection of H2O2 by immobilizing hemoglobin (Hb) on an MXene modified electrode [69]. The use of MXene lowered the LOD of the sensor to the nM range. A similar type of Hb-MXene based biosensor was reported for the electrochemical detection of nitrite using cyclic voltammetry [70]. Kai et al. modified the MXene surface with horseradish peroxidase (HRP) to fabricate an electrochemical biosensor for the detection of H_2_O_2_. The HRP enzyme was immobilized on an MXene/chitosan/GCE electrode, and the film demonstrated good electrocatalytic activity toward the reduction of H_2_O_2_. The amperometric biosensor displayed a wide linear range from 5 to 1650 μmol. L^−1^, LOD of 0.74 μmol. L^−1^ and good operation stability. The biosensor was successfully applied for the sensing of H_2_O_2_ traces in food samples [71].

MXene composites were also used to develop biosensors used for the ultra-sensitive detection of phenol in real water samples. These sensors are based on the use of the Tyrosinase (Tyr) enzyme entrapped within a chitosan composite that increased the adhesion of MXene-Tyr onto the GCE. The mechanism for phenol detection involves the oxidation of phenol by Tyr into the corresponding o-quinone. Afterward, the electrochemical reduction of the o-quinone producing polyhydric phenol is measured electrochemically. A noticeable increase in the current vs. time was observed with increasing the concentration of phenol. The sensitivity of Tyr-MXene-Chi/GC (414.4 mA M^−1^) was about 1.5 times higher than that of Tyr-Chi/GC (290.8 mA M^−1^). The LOD was 12 nmol L^−1^ while the linear range was from 5.0 × 10^−8^ to 15.5 × 10^−6^ mol L^−1^ [4].

Other sensor configurations reported the use of MXene as a transducer for the detection of organophosphorus pesticides (OPs). Zhou and his coworkers reported an acetylcholinesterase (AChE) based sensor for the detection of malathion using Ti_3_C_2_ nanosheets and chitosan as an immobilization matrix. The electrochemical behavior of the AChE/CS-Ti_3_C_2_/GCE biosensor was studied by cyclic voltammetry and electrochemical impedance spectroscopy (EIS). The biosensor displayed excellent performance against malathion with a LOD as low as 0.3 × 10^−14^ M [72]. In another report, the same analyte, i.e., malathion was detected by AChE inhibition, using Ag modified MXene as a transducer. The modification of MXene with Ag NPs amplified the electrochemical signal; a LOD of 3.27 × 10^−15^ M was reported in this case [73].

**Figure 5:**
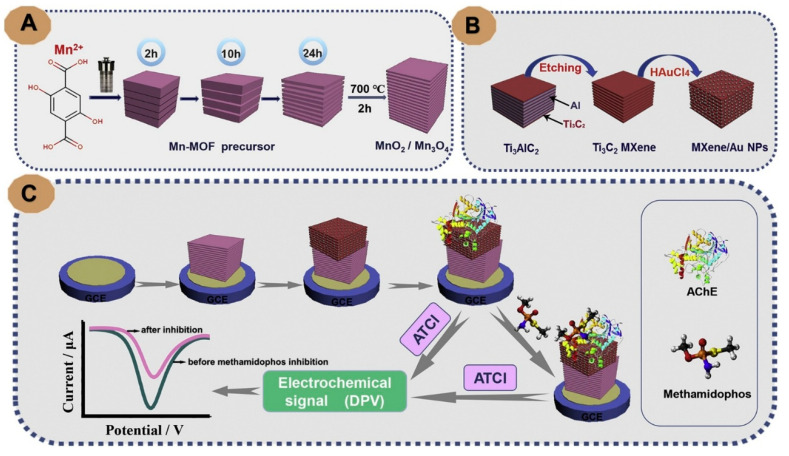
Illustration of the formation of MnO_2_/Mn_3_O_4_ composite (**A**), MXene/Au NPs (**B**), the fabrication process of AChE-Chit/MXene/Au NPs/MnO_2_/Mn_3_O_4_/GCE biosensor for methamidophos assay (**C**) (reproduced with permission from reference [74]).

Another configuration was adopted to design a biosensor for OPs by combining Ti_3_C_2_ MXene nanosheets with metal-organic frameworks (MOFs). The combined material, a MOF-derived MnO_2_/Mn_3_O_4_, Ti_3_C_2_ MXene/Au NPs composite, was used as an electrochemical biosensing platform with immobilized AChE enzyme, constructed as shown in Figure 5. The vertically aligned, highly ordered nanosheets of Mn-MOF derived 3D MnO_2_/Mn_3_O_4_ combined with MXene/Au NPs yielded a synergistic amplification effect, providing enlarged specific surface area and good environmental biocompatibility. Using this biosensor, the detection of methamidophos was achieved over a broad concentration range (10^−12^–10^−6^ M). TheLOD (1.34 × 10^−13^ M) of the biosensor far exceeds the maximum residue limits (MRLs) for methamidophos (0.01 mg/kg) established by European Union [74].

Applicability of several of these enzyme-based MXene biosensors has been tested for food quality analysis. A Ti_3_C_2_ based double enzyme biosensor was reported for the detection of inosine monophosphate (IMP), which imparts flavor to the meat and can be used as an indicator to assess meat quality [75]. The developed biosensor was based on a nanohybrid structure consisting of Ti_3_C_2_ MXene as a highly conductive and stable base material, modified with two enzymes (5′-nucleotidase and xanthine oxidase) and a core-shell bimetallic nanoflower composed of an Au core and a Pt shell. In this configuration, the MXene provided a stable biocompatible microenvironment for enzyme loading, while the double-enzyme was used to hydrolyze IMP to produce H_2_O_2_, which can be easily determined electrochemically through the bimetallic nanoflowers. Therefore, the content of IMP was indirectly quantified by monitoring the current change due to H_2_O_2_ production. The LOD achieved by this double enzyme biosensor was 2.73 ng mL^−1^ with a correlation coefficient of 0.9964 and a linear range between 0.04 and 17 g L^−1^.

### 3.2. MXenes Based Electrochemical Immunosensors

**Figure 6:**
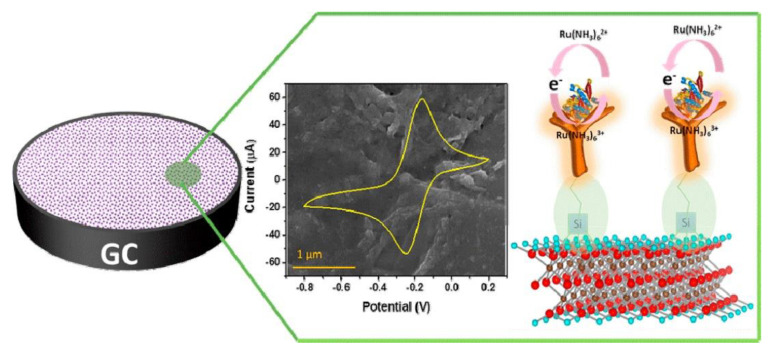
Schematic of the electrochemical carcinoembryonic antigen (CEA) detection mechanism (reproduced with permission from reference [62]).

Immunosensors combining immunochemical reactions based on the antibody (Ab)-antigen (Ag) recognition with an appropriate electrochemical transducer provide significant advantages for bioanalysis, including cost-effectiveness, low reagent and sample volume, portability, high-specificity and sensitivity, and high-throughput analysis [76]. Immunosensors have applications in various fields of analysis, such as clinical medicine [77,78], environmental pollutant evaluation [79,80,81], food inspection [82,83], and pathogenic microorganism detection [84,85]. The incorporation of MXenes in the fabrication of immunosensors provide opportunities for increasing bioactivity of immobilized Abs on electrode surfaces, increasing surface area, and improving detection sensitivity. Kumar et al. fabricated an immunosensor for the detection of carcinoembryonic antigen (CEA) [62], an important cancer biomarker found in the liver, breast, lung, colorectal, ovarian, and pancreatic cancer patients. To fabricate the sensor, amino-functionalized Ti_3_C_2_ MXene was used to chemically immobilize -COOH terminated-CEA. The electrochemical detection mechanism of the sensors is shown in Figure 6. The MXenes were synthesized using a layer delamination method that reduces the surface defects in the MXene and produces MXene nanoflakes with higher electrical conductivity. The biosensor showed a linear detection range of 0.0001 to 2000 ng mL^−1^ with a sensitivity of 37.9 µA ng^−1^ mL cm^−2^ and a stability up to 7 days. Recovery studies for the quantification of CEA in serum samples indicated promising results.

Another MXene based CEA biosensor has been reported using a sandwich-type immunoassay format in which Ti_3_C_2_ was first functionalized with amino silane (APTES) for covalent immobilization of monoclonal anti-CEA antibodies (Ab_1_) with surface plasmon resonance (SPR) detection. A MXene-hollow Au NPs (HGNPs) nanohybrid was synthesized and further decorated with staphylococcal protein A (SPA) to immobilize the polyclonal anti-CEA detection antibody (Ab2) and serve as signal enhancers. The capture of CEA resulted in the formation of an Ab2-conjugated SPA/HGNPs/N-Ti3C2-MXene sandwiched nanocomplex on the SPR chip and the generation of a response signal. This SPR immunoassay had a reported LOD of 0.15 fM and a linear range of 0.001 to 1000 pM.

**Figure 7:**
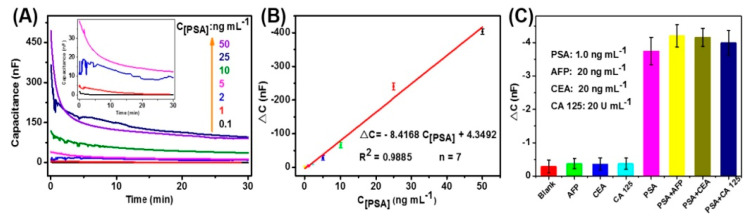
Analytical performance of the developed immunoassay (**A**) capacitance responses of Ti_3_C_2_ MXene-based interdigitated immunosensor toward target PSA standards; (**B**) the corresponding calibration plots; and (**C**) the specificity of capacitance immunosensor against target PSA and non-targets including AFP, CEA, and CA 125 (reproduced with permission from reference [86]).

In other configurations, Chen et al. developed a Ti_3_C_2_ MXene-based interdigitated capacitance immunosensor for the detection of prostate-specific antigen (PSA), an important biomarker for used to screen prostate cancer. The normal levels of PSA in the serum of healthy males are less than 4.0 ng mL^−1^, whereas rising levels are associated with prostate cancer. The biosensor utilized an MXene modified interdigitated micro comb electrode to immobilize anti-PSA capture Ab, whereas Au NPs functionalized with horseradish peroxidase (HRP) and detection antibody were used as the signal-transducer tags. To further increase the sensitivity of the assay, a tyramine signal amplification and enzymatic biocatalytic precipitation were coupled with the main immunochemical reaction. Under optimum conditions, the change of the immunosensor in the capacitance increased with the increasing target PSA concentrations from 0.1 ng mL^−1^ to 50 ng mL^−1^ at a detection limit of 0.031 ng mL^−1^. The characteristics of the developed system are shown in Figure 7. Despite these merits, one of the limitations of this immunosensor is the complex fabrication procedure and long reaction time as compared to commercial enzyme-linked immunosorbent assays [86].

### 3.3. DNA/Aptamer Based Biosensor

Biosensors with nucleic acid detection using either optical or electrical output to monitor the hybridization event have been widely reported as bioanalytical platforms for many applications [87]. Recently, MXenes have been explored as an electrode material to monitor the hybridization event and enhance detection sensitivity. Zheng et al. reported a DNA/Pd/Pt nanocomposite for the electrochemical detection of dopamine (DA) [88], an important catecholamine neurotransmitter in living organisms that plays an essential role in the function of human renal, metabolism, cardiovascular, and central nervous systems [89]. Abnormal DA levels may indicate neurological disorders and a variety of acute and chronic diseases such as Schizophrenia, Parkinson, and Alzheimer [90]. In the developed biosensor, MXene nanosheets act as a matrix for the loading of the DNA and Pd/Pt. First, the DNA was adsorbed on the MXene surface through π-π stacking interaction between the nucleotide bases and MXene nanosheets, then the Pd and Pt NPs were synthesized in-situ in the presence of DNA/MXene nanocomposite. The results revealed that the presence of DNA prevents the restacking of Ti_3_C_2_ nanosheets and facilitates the even growth of PdNPs and Pd/Pt NPs. Moreover, the deposition of Pd/Pt NPs onto Ti_3_C_2_ nanosheets enhanced the electrocatalytic activity of the nanocomposites towards DA. In the final step, a GCE electrode was modified with the DNA/MXene/NPs composite to create the DA biosensor. The amperometric biosensor exhibited DA detection capabilities in concentration range of 0.2 to 1000 μM with a LOD of 30 nM (S/N = 3) and high selectivity against uric acid, ascorbic acid, and glucose [88].

**Figure 8:**
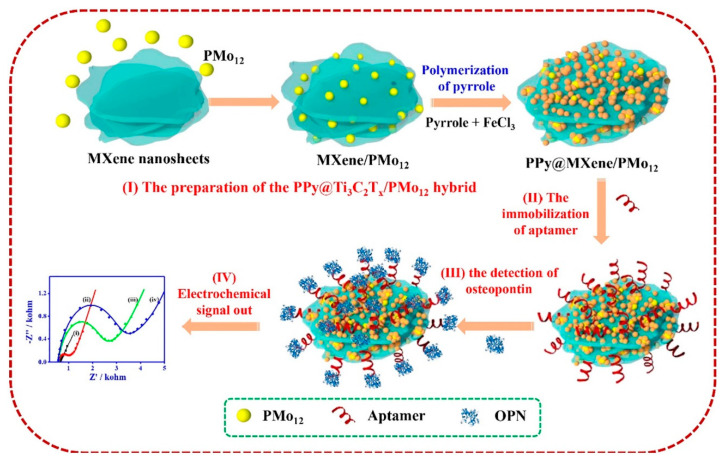
Schematic diagram of the aptasensor fabrication based on PPy@Ti_3_C_2_/PMo_12_ for the OPN detection. (reproduced with permission from reference [63]).

The detection of specific DNA sequences is significant, not only in clinical diagnostics but also in the environment and food analysis fields. Wang and his coworkers developed a nanobiosensor for gliotoxin [84], one of the most toxic mycotoxins produced by Aspergillus fumigatus, which poses a serious threat to humans and animals health. The biosensor was prepared by modifying MXene nanosheets with a tetrahedral DNA nanostructure (TDN), which acts as the main sensing element. The stated benefits of incorporating MXenes in the sensor design included increasing the sensitivity and providing an ample surface area for immobilizing a much greater amount of TDN onto the electrode. Moreover, the titanium element on the surface of MXene nanosheets offers a facile method for assembly via strong chelation interaction between the titanium and phosphate groups of TDNs, thus eliminating the need for complex and costly chemical modification of TDNs, which is generally required for the immobilization of TDNs onto the electrode. The amperometric response of the optimized biosensor responded to gliotoxin concentrations increasing from 5 pM to 10 nM, with an LOD of 5 pM. Zhoe et al. reported an impedimetric aptasensor for electrochemical detection of osteopontin (OPN), a cancer biomarker that is also responsible for tumor growth and progression in human cervical cancer. The aptasensor was based on Ti_3_C_2_ MXene-phosphomolybdic acid (PMo_12_)-polypyrrole (PPy) nanohybrid used as an immobilization matrix for the anti-OPN aptamer. The fabrication procedure of the developed aptasensor is represented in Figure 8. The PPy@Ti_3_C_2_/PMo_12_-based aptasensor exhibited high selectivity and stability along with an extremely low detection limit of 0.98 fg mL^−1^, and applicability in human serum samples [63].

Apart from its use as transducer surface in electrochemical biosensors, MXenes have also been used as bioimmobilization material or quencher in optical DNA assays. For example, Peng et al. designed an “off-on” fluorescent biosensor for the detection of Human papillomavirus (HPV) infection [91]. HPV is a human pathogen known to induce cervical cancer, the second most common cancer in women [92]. The assay was designed based on a fluorescence quenching mechanism, which has already been used in various fluorescence-based biosensors [93]. A fluorescent dye (FAM) labeled ssDNA was used as a fluorescent probe while Ti_3_C_2_ MXene was used as a nanoquencher. In the presence of Ti_3_C_2,_ the fluorescence of the FAM tagged ssDNA probe (P) was completely quenched while after interacting with its complementary DNA target (T), a P/T hybridized dsDNA structure is formed, which resulted in the recovery of the fluorescence signal. Furthermore, Exonuclease III (Exo III) was used to improve the sensitivity by enhancing fluorescence. Interestingly, when Exo III was introduced in the hybridization process, the 3′ end of the newly formed dsDNA was recognized by the Exo III, and then the hydrolysis of the P/T complex was initiated. This cycling process facilitated by the Exo III enhanced the fluorescence of P/T lowering the limit for HPV-18 detection to 100 pM [91].

**Figure 9:**
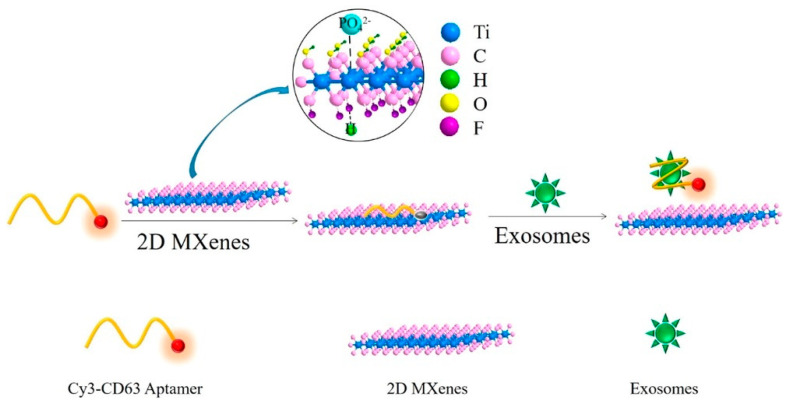
Schematic illustration of the developed fluorescence resonance energy (FRET)-based aptasensor (reprinted with permission from reference [94]).

Utilizing MXenes as a nanoquencher, Zhang et al. designed a fluorescence resonance energy transfer (FRET) bioassay for the detection of exosomes (Figure 9). This platform is based on a Cy3 dye-labeled CD63 aptamer (Cy3-CD63 aptamer)/Ti_3_C_2_ MXenes nanocomplex in which the fluorescence of the dye was quenched by the MXene nanosheets, while the addition of exosomes in the reaction mixture immediately recovered the fluorescence. The turn-on fluorescence phenomenon was ascribed to the release of the Cy3-CD63 aptamer from the surface of MXenes because of the relative strong specific recognition between the aptamer and the CD63 protein on exosomes. The assay achieved a LOD 1.4 × 10^3^ particles mL^−1^, which is reported as 1000× lower than the conventional ELISA based assay [94].

### 3.4. MXene for Next Generation Wearable Biosensors

Wearable electronics attracted considerable attention since the commercialization of smartphones and other portable health monitoring devices due to their ability to provide substantial information regarding the health of an individual. Early research in this field focused on the development of physical sensors that could monitor the heart rate, oxygen level, movement such as steps and calories burned, etc., but in the last years, the focus broadened to tackle challenges in health care applications such as monitoring of disease biomarkers. The high specificity, portability, and low power consumption of wearable biosensing devices hold promise for such applications. These include biosensor platforms for noninvasive analysis of biofluids, such as interstitial fluid (ISF), sweat, saliva, or tears. These fluids have been targeted mostly because of the advantage of noninvasive sampling, minimizing the risk of infection during the sampling procedure and user-friendly operation.

Key characteristics when designing wearable devices are the flexibility, mechanical properties, and conductivity of the material used as a sensing platform. Challenges include the integration and scalability of the bioelectronics components in order to achieve the maximum performance in an accurate and sensitive manner and ensure manufacturability. Large surface area nanomaterials have been explored as materials for flexible sensors to increase the effective contact area and impart favorable electrical and mechanical properties. MXenes represent a new wave of materials for wearable sensors and biosensors. The main applications explored thus far include pressure and strain sensors [95], piezoresistive sensors [96], and chemical sensors for detection of subtle pressure, simultaneous monitoring of human activities, quantitative illustration of pressure distribution, human-computer interaction, and electronic skin. The incorporation of MXenes in conjunction with biomolecules for wearable applications is relatively unexplored but rapidly emerging.

Cheng et al. used MXene to fabricate a piezoresistive sensor with spinous microstructures inspired by the human skin, which can be considered a sensitive biological sensor. The sensor was prepared using a simple abrasive paper stencil printing method. The spinous microstructures effectively increased contact area of the conductive channels, consequently improving the performance of the pressure sensor: Low-pressure detection limit (4.4 Pa), fast response time (<130 ms), high sensitivity (151.4 kPa^−1^), and excellent stability over 10,000 cycles [97]. Guo et al. presented a similar kind of flexible, highly sensitive, and degradable pressure sensor. A porous MXene-impregnated tissue paper was sandwiched between an interdigitated electrode-coated PLA thin sheet and a biodegradable polylactic acid (PLA) thin sheet. The flexible sensor exhibited high sensitivity (10.2 Pa), fast response (11 ms), low power consumption (10–8 W), biodegradability, and excellent reproducibility over 10,000 cycles [98]. Li and Du reported an MXene-Ag nanocomposite based sensitive strain sensor. The sensor is based on 1D Ag nanowires, 0D Ag NPs and 2D MXene nanosheets. The Ag NPs act as a bridge between the MXene sheets and the Ag nanowires. This design helped to increase the elasticity and conductivity of the sensor. This fabric-based strain sensor was composed of elastic textile material (double-covered yarn) that was doped and blended with Ag/MXene nanocomposite, creating a wearable clothing material. The as-prepared sensor showed highly sensitive and stretchable performance with a high gauge factor at an exceptionally large strain (350%). In addition to wearable textiles, the developed sensor has potential applications in biomedical and fire safety applications [99]. Other configurations use MXene in conjunction with hydrogels to produce wearable sensors [100,101].

**Figure 10:**
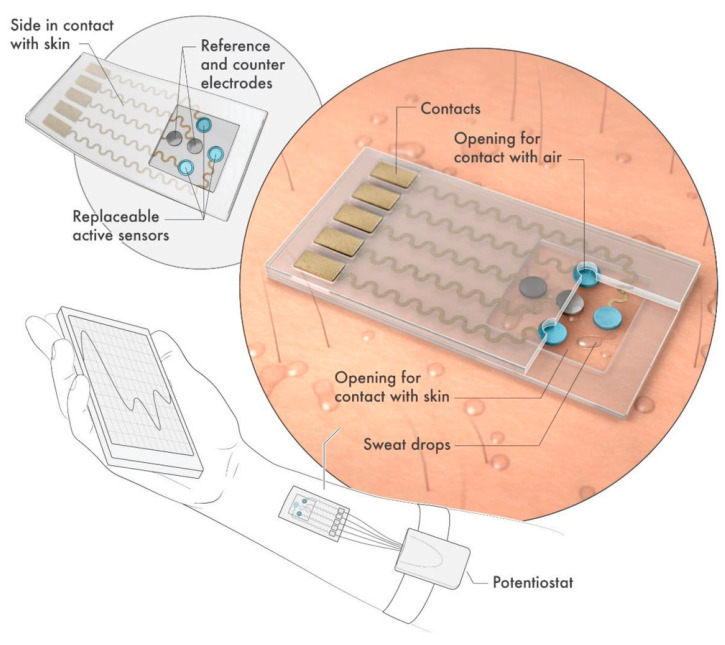
Schematic diagram of wearable sweat sensor (reprinted with permission from reference [102]).

Apart from these physical sensors, Lei et al. have developed an MXene-based wearable biosensor for sweat analysis. The device was fabricated by using 2D MXene (Ti_3_C_2_T*_x_*) nanosheets as material to develop an oxygen-rich enzyme biosensor for H_2_O_2_ detection based on a Ti_3_C_2_T*_x_*/PB (Prussian Blue) composite and enzyme. Due to the high conductivity and excellent electrochemical activity of exfoliated MXene, Ti_3_C_2_T*_x_*/PB composites showed better electrochemical performance compared to carbon nanotubes/PB and graphene/PB composites toward H_2_O_2_ detection. The sensing device contains a versatile replaceable sensor component, which can be inserted and changed with customized sensors prepared to track different analytes, i.e., glucose, lactate, or pH value (Figure 10). The substrate of the device was composed of superhydrophobic carbon fiber that was used to create a tri-phase interface and protect the connector from sweat corrosion. The sensing performance of the as-prepared device was evaluated using artificial sweat. Electrochemical results showed sensitivities of 11.4 µA mm^−1^ cm^−2^ for lactate 35.3 µA mm^−1^ cm^−2^ for glucose. Furthermore, the sensor was tested on human subjects and used for sweat analysis. The obtained results showed simultaneous measurements of glucose and lactate with high sensitivity and repeatability [102].

## 4. Conclusions and Future Prospects

In summary, MXenes are an emerging class of 2D nanomaterials mainly composed of transition metal carbides, nitrides, or carbonitrides. MXenes have gained substantial attention in fields such as batteries and supercapacitors, and their application in chemical and biological sensors is growing. This paper provided an overview of the MXenes properties and their incorporation in the development of enzyme, antibodies, and aptamer-based bioanalytical sensors. As compared to the previously discovered 2D nanomaterials, MXenes possess some superior characteristics such as hydrophilicity, ease of functionalization, high electron transport capability, and vast compositional variety, high surface area due to their “accordion-like” morphology, and biocompatibility, which makes them attractive for biosensing applications. The surface terminal functional groups such as -O, -OH, -F are amenable to functionalization with biomolecules and enable the development of innovative bioanalytical platforms. A growing area is to incorporate MXene in wearable technologies and devices that have the required stability, flexibility, and targeting properties to be interfaced with the human body. This area is expected to grow in the future with new developments in processing, integration, and manufacturing.

Several challenges need to be addressed in order to utilize the full potential of this novel class of materials. (1) Current synthetic methods to create MXenes involve the use of HF as an etchant. The method is not environmentally-friendly and it cannot be easily scaled up, thus hindering production at a commercial scale. Other mild etchants such as LiF/HCl, molten ZnCl_2_ have also been reported, but the final yield is very low. Significant progress is needed to advance the sustainable and scalable synthesis of MXenes. Moreover, a large number of MXenes have been predicted theoretically, but only a few have been actually produced by exfoliating MAX phases. Thus, it is important to shift the synthesis procedure from theoretical prediction to wet chemical labs. (2) The HF based etching method produces MXenes with randomly functionalized surfaces and it is difficult to obtain MXenes with specific and uniformly distributed surface termination. (3) The presence of fluorine on the surface hinders the use of MXenes in biomedical applications or for the immobilization of biomolecules. Therefore, new fluorine-free synthesis routes with high yield need to be developed, or these groups should be removed from the surface. (4) The top-down synthesis gives less control over tuning the morphology and surface modification of the MXenes. Hence, a bottom-up synthesis approach of MXenes should be developed as an alternative. (5) The long-time sonication for delamination produces MXenes with high surface defects that could possibly alter the properties of MXenes. Additionally, organic solvents are being used for intercalation and delamination, which is also not a very environmentally friendly approach. (6) Lastly, the oxidation of MXenes in oxygenated solvents is a major obstacle. Other approaches are needed to make it possible to use MXenes in the anodic potential window without compromising the inherent physicochemical properties of MXenes. Looking ahead, more effective synthesis and surface modification strategies are required to advance application of MXene in the development of practical bioanalytical sensors and other applications. Scalability in synthesis and processing as well as integration and more effective transduction, are needed to ensure future growth and implementation of these materials in wearable sensing applications.

## Figures and Tables

**Table 1:** Comparison of fundamental properties of various nanomaterials.

Nanomaterials	Surface Area (m^2^ g^–1^)	Conductivity (S/cm)	Band Gap (eV)	Biocompatibility	References
Graphene	450	2700	0	Biocompatible	[14,15,16]
h-Boron nitride	150–550	insulator	5.9	Dependent on size and shape	[17,18]
SWCNT	600	10^2^–10^6^	0.042	Unclear /under debate	[19,20,21]
MWCNT	122	10^3^–10^5^	1.82	Unclear/under debate	[19,20,22]
MoS_2_	8.6	10^−4^	1.89	Biocompatible	[23,24,25]
δ-MnO2	257.5	10^−5^ to 10^−6^	1.33	Biocompatible	[15,26,27]
MXene (Ti_3_C_2_)	93.6	2410	0.1	Biocompatible	[28,29,30]
